# Hyperbaric Oxygen Therapy for Soft Tissue Injury in Open Musculoskeletal Trauma: A Prospective Study

**DOI:** 10.7759/cureus.48848

**Published:** 2023-11-15

**Authors:** Manish Raj, Raj K Bhartiya, Ajay K Rajput, Santosh Kumar Singh, Jitendra Pal Singh Jadon, S. P. S. Gill

**Affiliations:** 1 Orthopedics, AIIMS Deoghar, Deoghar, IND; 2 Orthopedics and Traumatology, Maa Vindhyawasini Autonomous State Medical College, Mirzapur, IND; 3 Orthopedics, Uttar Pradesh University of Medical Sciences, Etawah, IND; 4 Department of Orthopedics, Government Medical College, Kannauj, Kannauj, IND

**Keywords:** open fracture, traumatic wounds, conventional wound dressings, musculoskeletal trauma, hyperbaric oxygen treatment

## Abstract

Background

Non-union, chronic pain, functional disability, and infection are all things that have been associated with open fractures with severe soft tissue damage leading to the need for additional hospitalization, and sometimes even subsequent surgeries and weeks or months of rehabilitation. Open fractures and severe musculoskeletal injuries are occasionally treated with hyperbaric oxygen therapy (HBOT) in an effort to reduce the risk of complications and increase the likelihood of a successful recovery.

Methods

A prospective randomized controlled study was done between January 2019 and August 2022 at a tertiary health care center including 60 patients with a severe soft tissue injury (Grade II and III) divided into two groups - group-CT (30 patients who received conventional treatment) and group HT (30 patients, who received HBOT in addition to conventional treatment). The outcome was measured according to the Bates-Jensen Wound Assessment Tool.

Results

The wound size, depth, and granulation were significantly reduced in group-HT patients. In the final session, the patient’s severity of the wound in group-HT was significantly reduced (P = 0.0001) compared to group-CT.

Conclusions

Patients who received HBOT reported a significant improvement in their wounds.

## Introduction

In addition to the pain, cost of hospitalization, and functional disability that follow musculoskeletal trauma, complex open fractures with severe soft tissue injury have been associated with late complications like non-union of the fractured bone, deep infection, and delayed union that frequently necessitate multiple additional interventions [1-3].

With regard to such injuries, hyperbaric oxygen therapy (HBOT) has beneficial therapeutic effects, such as anti-infective effects that are complementary to or synergistic with antibiotics, decreases in edema and ischemic necrosis, decreasing reperfusion injury, and the potential to hasten fracture healing and soft tissue healing by reducing tissue edema (vasoconstriction and improved homeostasis) and stimulating angiogenesis [3-7]. In the context of orthopedic and soft-tissue trauma, all of these measures are appealing [8].

Various adjunctive treatment modalities including HBOT, and cryotherapy are being evaluated for use in clinical practice to treat musculoskeletal problems; however, HBOT is thought to show the most promise as a useful supplementary treatment for muscle repair, reduce the incidence of soft tissue infection, and reduce the late complications of open fractures like delayed union [9-11].

In HBOT, 100% oxygen is administered at pressures greater than one absolute atmosphere (ATA) for therapeutic purposes. Patients are placed in a pressured chamber (either a multiplace or monoplace-one man) for 60-120 minutes, once or twice daily, at a pressure of 1.5-3.0 ATA [12]. With proper pre-exposure orientations, complications such as oxygen intoxication, barotrauma to the middle ear, and solitary confinement anxiety are well controlled [13]. Although HBOT has shown promise in promoting recovery from skeletal muscle injury, its potential to do so mechanistically remains unproven, and the therapy is not yet accepted for use in clinical practice [14].

However, various animal experimental studies have shown the promising results of HBOT on muscle injuries there are few studies available in the literature to show the effect of HBOT on open complex musculoskeletal trauma in human beings [9,15,16]. So, we designed a prospective study to evaluate the role of HBOT in patients diagnosed with open fractures associated with severe soft tissue injury.

## Materials and methods

A prospective randomized controlled study was done between Jan 2019 to August 2022 at a tertiary health care centre situated in rural area of north India, after obtaining clearance from Institutional Ethical Committee of Uttar Pradesh University of Medical Sciences (UPUMS/Dean/M/Ethical/1365/182/2018).

All patients above 18 years of age who had suffered an open musculoskeletal trauma with severe soft tissue injury (Grade-II and III) and presented to hospital within three days of injury are included in this study. However, all patients suffering from polytrauma, chest injury, comorbid medical conditions, psychiatric disorders, peripheral vascular disease and pregnancy were excluded from this study.

**Figure 1:**
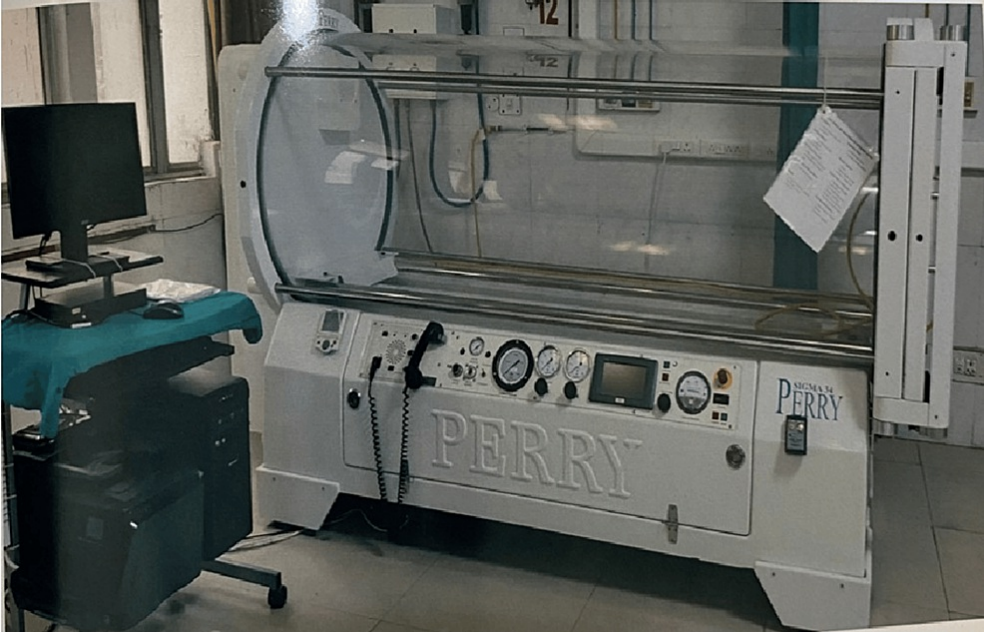
Monoplace hyperbaric chamber

A total of 60 patients were included in this study which were divided into two groups based on treatment provided. Patients were allocated in each group by using random technique (lottery method) and we had 30 participants in each treatment group. In one group only convention therapy (Group-CT) in the form of different types of regular dressing, e.g., simple gauze, betadine solution, hydrogen peroxide, eusol solution along with and local debridement at bedside or in the operating room was done. In another group (Group-HT), different sessions of HBOT in addition to convention therapy was given with the help of monoplace-chamber (Figure 1).

Duration of treatment in both the groups depend on wound condition. HBOT was provided using pressure/time schedule delivering a dose approximately bioequivalent to 90 min of oxygen breathing at 2.4 atmospheres absolute pressure. Treatments were delivered twice daily for the first three days afterward on daily basis.

Patients in the HT group were planned to undergo a total of 12 HBOT sessions over the course of about nine days. If a patient was discharged from the hospital before the expected 12 HBOT sessions with a healed wound, then the patient was regarded to have completed a full course of treatment notwithstanding the number of HBOT sessions received.

A complete blood count was taken, as well as a urine sample, a kidney function test, and an examination of the patient's chest x-ray. The length of time a patient had a wound-related medical condition was noted, as was the patient's smoking and hypertension histories, as well as their current and past treatments.

Initial trauma evaluations were performed on all patients using ATLS guidelines, and individuals with more serious life-threatening injuries were excluded. After the patient was resuscitated and stabilized, limb salvage and functional restoration began. Intravenous broad-spectrum antibiotics, irrigation, rigorous debridement, stable bone fixation, repair of specialized tissue like nerve and tendon, and early definitive soft tissue coverage were the cornerstones of wound management.

Local examination of the wound for infection and the process of healing was be done using Bates-Jensen Wound Assessment Tool (BWAT) [17]. Size (length and width), number, type of wound, infection and discharge was recorded. Wound characteristics was analysed on first, and final sessions (depend on wound status) and assessment of healing in both the groups were noted on the basis of BWAT as outcome. The requirement of secondary soft tissue procedure was also recorded at final session.

Statistical analysis

The statistical analysis has been performed using SPSS 22 software version (IBM Corp., Armonk, NY). The statistical analysis of quantitative data (mean ± SD) between the groups was done by student “t” test. The statistical analysis of qualitative data (N, %) between the groups is done by using Chi-square test. P < 0.05 was considered significant.

## Results

The demographic details of patients of both groups are shown in Table 1.

**Table 1:** Demographic details of the patients. ^1^Data were presented in Mean ± Standard deviation.

S.N.	Variables		Hyperbaric Oxygen therapy (Group-HT), N (%)	Conventional therapy group (Group-CT), N (%)	P-value
1	Age (year)	18 - 40	5 (16.66)	6 (20)	>0.05
		40 - 60	20 (66.66)	18 (60)	
		>60	5 (16.66)	6 (20)	
2	Sex	male	20 (66.66)	19 (63.33)	
		female	10 (33.33)	11 (36.66)	
3	Mechanism of injury	Road traffic accident	25 (83.33)	24 (80)	
		Fall from height	5 (16.66)	6 (20)	
4	Time duration from injury to hospitalization (hours)	<24	18 (60)	14 (46.66)	
		24 - 48	8 (26.66)	8 (26.66)	
		>48	4 (13.33)	6 (20)	
5	Classification of Wound (According to Gustilo Anderson)	III A	6 (20)	3 (10)	
		III B	24 (80)	27 (90)	
6	Primary management of fracture	External fixator	15 (50)	18 (60)	
		Intramedullary nailing	10 (33.33)	8 (26.67)	
		Plating	5 (16.67)	4 (13.33)	
7	Length of stay in hospital (days)^1^		20.7 ± 5.07	39.56 ± 8.15	0.0001

A total of 60 patients were randomly allotted in two groups. Group HT included 30 patients who received HBOT in addition to conventional therapy, and group CT was the control group, which included 30 patients who received only conventional therapy. In demographic details, there was no significant difference found in both groups regarding the age of the patient, sex, mechanism of injury, time duration from the event to hospitalization, type of wound, and the type of primary fracture fixation. However, the average hospital length of stay among participants in both groups was found to be statistically significant in correlation favoring group HT (P = 0.0001). Table 2 shows BWAT evaluation at the first session and final session.

**Table 2:** Wound assessment (Bates-Jensen Wound Assessment Tool) evaluation at first session and final session. ^1^Data were presented in N (%).

S.N.	Variables		At first session			At final session		
			Hyperbaric Oxygen therapy group (Group-HT) (Mean±SD)	Conventional therapy group (Group-CT) (Mean±SD)	P-value	Hyperbaric Oxygen therapy group Group-HT (Mean±SD)	Conventional therapy group (Group-CT) (Mean±SD)	P-value
1	Size (cm^2^)		64.7 ± 23.51	54.56 ± 20.97	0.08	10.46 ± 3.05	15.2 ± 5.6	0.0002
2	Depth (mm)		12.9 ± 3.26	12.1 ± 2.7	0.30	1.23 ± 0.43	1.7 ± 0.59	0.001
3	Granulation (Grade)		4.8 ± 0.40	4.7 ± 0.46	0.37	2.06 ± 0.86	2.96 ± 0.80	0.0001
4	Severity^1^	Non-severe	3(10)	0	0.959	26(86.66)	5(16.66)	0.0001
		Severe	27(90)	30(100)		4(13.33)	25(83.33)	
5	Infection^1^	Present	24(80)	21(70)	0.37	3(10)	10(33.33)	0.028
		Absent	6(20)	9(30)		27(90)	20(66.67)	

The BWAT evaluation at the first session showed a similar distribution of size (cm^2^), depth (mm), and granulation in both groups. However, The BWAT evaluation at the final session showed significantly higher mean ± SD of size, depth, and granulation in CT-group than in HT-group (P < 0.05). At the time of admission, the majority of patients had infection in both the HT and CT groups (P = 0.37); however, at the final session, the majority of patients had no infection in either the HT or CT groups (P = 0.052). At the final session, the patient’s severity of the wound in group HT was reduced to 13% from 90%, while in the CT group, no such changes were seen (P = 0.0001). In final session, in the HT-group majority of patients 50% not required any secondary procedure compared to CT-group in which 50% required a skin graft and 20% required a skin flap (Table 3).

**Table 3:** Requirement of secondary soft tissue procedure in both groups at the final session.

Types of soft tissue procedure required	Hyperbaric Oxygen therapy (Group-HT), N (%)	Conventional therapy group (Group-CT), N (%)	P-value
Not required	15 (50)	9 (30)	0.24
Skin Graft	12 (40)	15 (50)	
Skin Flap	3 (10)	6 (20)	

Less secondary intervention was required in group HT-group than CT-group, but statistically not significant due to the small size of the sample (P = 0.24).

## Discussion

HBOT is effective for primary and secondary treatment and is becoming increasingly appealing for various purposes due to novel and established pathophysiological effects. Although we did not find a statistically significant decrease in the need for secondary intervention for wound closure in the patients who received HBOT sessions in the current trial, in individuals who completed HBOT sessions, the severity of the wound considerably lessened.

Using a rat skeletal muscle damage model, Oyaizu et al. [18] treated contused calf muscles with 100% oxygen at 2.5 atmospheres absolute for two hours daily for five days following injury. By inhibiting acute macrophage elevation and then speeding up macrophage invasion into the contused muscle, by increasing the number of proliferating and differentiating satellite cells, and by increasing the amount of regenerated muscle fibers by stimulating the IL-6/STAT3 pathway, HBOT decreased early lower limb volume and muscle wet weight in contused muscles and increased muscle isometric strength seven days after injury. Finally, they observed that HBO serves a double role in the healing process following muscle contusion injuries by both reducing inflammation and accelerating myogenesis.

In order to increase the functional outcome and lessen limb loss in 45 patients with crush injuries, Chiang et al. [19] examined the use of HBOT as an additional therapy in mangled hand injuries. Patients underwent 120 minutes of HBOT with oxygen at 2-5 atmospheres absolute while breathing 100% oxygen after reconstruction or revascularization. The average number of HBOT sessions was 9.1 (range, 6-14), and the outcomes were amputee survival and complications from the surgery. According to their findings, 81% of the replanted fingers and 100% of the replanted palms survived. The first reconstruction's difficulties included the partial loss of an avulsed flap, while the majority of the chronic stage's (three-month) complications involved scar contracture. They came to the conclusion that early intervention utilizing supplementary HBOT was successful in maintaining partially viable tissue and restoring hand function in patients with a mangled hand injury when used in conjunction with careful microsurgery.

HBOT was linked to an increase in blood vessel growth and viability in random rat skin flaps in an experimental study by Rech et al. [20] to evaluate the impact of HBOT on angiogenesis in rat skin flaps by immunoexpression of vascular endothelial growth factor A (VEGF-A). This finding paves the way for further research to confirm and clarify the mechanism of action.

By randomly assigning the 120 patients with open tibial fractures within 48 hours of injury to receive standard trauma care or standard care plus 12 sessions of HBOT, Millar et al. [9] were able to reduce complications and improve outcomes in their international multi-center randomized clinical trial on the use of HBOT in the management of open fractures and severe soft tissue crush injuries. The incidence of necrosis, infection, or both occurring within 14 days of the injury was documented as the main outcome. They found that 29% of HBOT patients experienced tissue necrosis, compared to 53% of controls (P = 0.01), and that HBOT patients experienced fewer late complications (P = 0.007), such as delayed fracture union (P = 0.04), and had better quality of life measures at one and two years. They ultimately came to the conclusion that early HBOT improves functional results and lowers tissue necrosis and the possibility of long-term consequences in severe lower limb damage.

Eskes et al. [21] conducted a Cochrane review of randomized controlled trials (RCTs) comparing HBOT with other therapies such as dressings, steroids, sham HOBT, or comparisons between other HBOT regimens to assess the efficacy of HBOT on the healing of acute surgical and traumatic wounds. Only three trials satisfied the authors' criteria for inclusion in the review, despite the extensive search. They were unable to definitively prove or disprove the efficacy of HBOT due to a lack of sufficient RCTs. Their analysis was limited by the studies' small sample sizes and the lack of comparable data. The Cochrane study group suggested conducting additional high-quality, RCTs for acute surgical and traumatic wounds, with endpoints like mortality, pain scores, quality of life, patient satisfaction, activities of daily living, length of hospital stay, and costs, that are powered to meet FDA guidelines. The development of evidence-based guidelines for the treatment of crush injuries remains a challenge for the practice of HBOT as a whole.

A small sample size and a short-term follow-up were the main limitations of the study. We only focused on the presence of wound infection and the wound healing process as the primary outcomes in the final session. It is unclear how many sessions of HBOT are necessary, and the optimal amount might change with injury severity. A study consisting of long-term follow-up with a large sample size assessing the final outcome as bony union, soft tissue complications, and infection is required.

## Conclusions

We did not find any evidence that patients who got HBOT sessions required less secondary intervention for wound healing in our trial. Patients who got HBOT, on the other hand, saw a considerable reduction in wound severity. The biological implications of HBOT and the efficiency of wound closure in complex musculoskeletal injuries will continue to be explored in future research. HBOT will become more affordable and convenient as it becomes more accessible to institutions, improving the chances for patients with crush injuries.
